# No ageing‐related increase in fibre type grouping in sprint‐trained masters runners: A 10‐year follow‐up study

**DOI:** 10.1002/jcsm.13416

**Published:** 2024-01-16

**Authors:** Guy Anselme Mpaka Messa, Marko T. Korhonen, Hans Degens

**Affiliations:** ^1^ Higher Institute of Medical Technology ISTM‐Kinshasa Kinshasa Democratic Republic of Congo; ^2^ Faculty of Medicine University Kasa‐Vubu (UKV) Boma Democratic Republic of Congo; ^3^ Faculty of Medicine University de Bandundu (UNIBAND) Bandundu Democratic Republic of Congo; ^4^ Gerontology Research Center, Faculty of Sport and Health Sciences University of Jyväskylä Jyväskylä Finland; ^5^ Department of Life Sciences, Institute of Sport Manchester Metropolitan University Manchester UK; ^6^ Institute of Sport Science and Innovations Lithuanian Sports University Kaunas Lithuania

**Keywords:** ageing, longitudinal study, masters athlete, motor unit

## Abstract

**Background:**

Previous research suggests that an ageing‐associated remodelling and loss of motor units due to motor neuron death contributes significantly to muscle weakness in old age. In histological sections, motor unit remodelling is reflected by increased fibre type grouping. While regular exercise may not attenuate the loss of motor units during ageing, it has been suggested to facilitate reinnervation resulting in larger motor units, and a higher number and larger fibre type groups in histological sections of muscles from aged individuals.

**Methods:**

In a 10‐year follow‐up study, we assessed changes in the prevalence and size of fibre type groups in the vastus lateralis muscle from 34 male masters sprinters (40–85 years at start).

**Results:**

Over the 10 years, there was an ageing‐related reduction in performance in the 60‐m sprint (*P* < 0.001) without significant changes in fibre type composition and fibre cross‐sectional area. Neither the number of fibre type groups, defined as a fibre surrounded exclusively by fibres of the same type, nor the group size changed significantly in the 10‐year period.

**Conclusions:**

These histological data show that there is limited to no significant fibre type grouping over a 10‐year period in masters athletes who continued sprint run training. This observation challenges the paradigm that ageing, at least in systematically trained sprinters, is associated with motor unit remodelling.

## Introduction

From as early as the age of 30 years, human ageing is characterized by a gradual decline in muscle mass,^1^ ultimately resulting in physical impairments, increased fall risk, reduced quality of life and loss of independence in older people.^2^ The ageing‐related loss of muscle mass and strength is thought to be at least partly due to the loss of motor neurons with consequent denervation of their associated fibres and ultimately disappearance if they are not reinnervated.^1^ Fortunately, most fibres that become denervated due to motor neuron loss are reinnervated by the branching axon of a viable motor neuron nearby. The reinnervated fibre then acquires the properties of the other fibres innervated by that motor neuron, which may result in fibre type grouping.^3^ Electrophysiologically, this is seen as a loss of motor units and an increase in motor unit size.^4^, ^5^

Lack of neuromuscular stimulation due to disuse is accompanied by muscle wasting and weakness. It has previously been suggested that most of the changes in skeletal muscle commonly seen in older adults are attributable to lower physical activity levels in old age, while regular exercise may attenuate the reduction in muscle function associated with typical ageing.^6^ In line with the benefits of regular exercise for the neuromuscular system, several studies have reported that vigorous exercise sustained for decades (e.g., training as performed by track and field masters athletes) may protect against the ageing‐related loss of motor units observed in inactive older individuals^7^ and/or promote reinnervation of muscle fibres as manifested electrophysiologically by larger motor units than in age‐matched non‐athletes^8^ and a higher prevalence of fibre type groups in histological sections.^9^

While the data of motor unit loss during ageing appear compelling, there is large variation in the number of motor units within a muscle between people of the same age.^4^, ^5^ It could be that part of the ageing‐related reduction in motor units and motor neurons observed in cross‐sectional studies may not be a real loss but rather a lower number of motor units at birth due to differences in lifestyle (e.g., pre/post‐natal diet) between people over the previous decades of their life. It has been shown that muscles in offspring from both maternal rats on a cafeteria diet^10^ or nutrient restriction^11^ have fewer fibres that may result in a lower motor unit number, as motor neuron survival (and hence motor unit number) during embryonic development is dependent on the number of muscle fibres available for innervation.^12^ In addition, one rodent study did not observe any ageing‐related reduction in the number of motor neurons.^13^ Moreover, a human study found that all ageing‐related muscle atrophy was explained by fibre atrophy rather than fibre loss.^14^ Furthermore, we recently observed no ageing‐related changes in the prevalence and size of fibre type groups, and any grouping that was observed was similar to that predicted by the fibre type composition.^15^ These findings suggest that motor unit remodelling may not have occurred in either ageing athletes or non‐athletes. Interestingly, the larger prevalence of fibre type grouping reported by Mosole et al.^9^ may not necessarily reflect better reinnervation but rather be a consequence of a larger proportion of type I fibres typically observed in masters endurance athletes.

All previous studies examining fibre type grouping and electrophysiologically determined motor unit remodelling in masters athletes were cross‐sectional. Thus, to ascertain the effect of ageing on motor unit loss and remodelling, longitudinal studies are required. To our knowledge, no study to date has examined changes in fibre type grouping in masters athletes longitudinally. We had the unique opportunity to examine changes in fibre type grouping in vastus lateralis muscles of 40‐ to 85‐year‐old (at the start of the study) masters sprinters over a 10‐year period.

## Materials and methods

### Study design and subjects

Thirty‐four male sprint‐trained masters athletes provided a muscle biopsy in both 2002 (when they were 40–85 years old) and 2012. They were recruited by means of personal letters from members of Finnish track and field organizations as previously described.^16^ The best performances in the 60‐m sprint in 2002 and 2012 were registered, and the number of weekly training hours and the number of years trained were estimated by using a questionnaire.^16^ For each participant, the body mass and height were measured, and the body mass index (BMI; kilograms per square metre) was calculated. The study was approved by the ethics committee of the University of Jyväskylä (Finland) and has been performed in accordance with the ethical standards laid down in the 1964 Declaration of Helsinki and its later amendments. All participants gave written informed consent.

### Muscle biopsy and histochemistry

Muscle biopsies were taken from the middle portion of the vastus lateralis muscle at 40% of the distance from the patella to greater trochanter under aseptic conditions by using either a conchotome or Bergström needle under local anaesthesia with 1–2% lidocaine, as described previously.^15^, ^16^ The specimen was mounted in embedding medium (OCT, Scigen®, Gardena, CA, USA), frozen in isopentane cooled in liquid nitrogen and stored at −80°C for later sectioning and staining.

**Figure 1:**
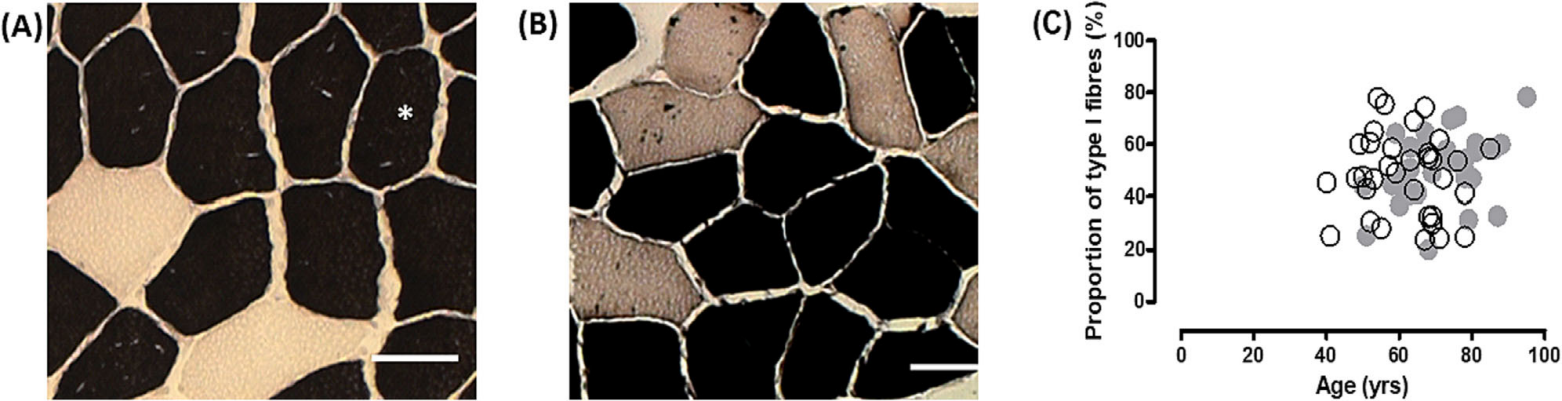
Examples of *m. vastus lateralis* biopsy cross sections obtained from a participant in (A) 2002 (54‐year‐old) and (B) 2012 (63‐year‐old) and stained for myosin ATPase after pre‐incubation at pH 4.30. Type I fibres were stained black, and type II fibres were stained light. *An enclosed type I fibre. Scale bar = 50 μm. (C) Proportion of type I fibres in 2002 (○) and 2012 (●).

For histochemical analysis, serial 10‐μm cross sections were cut on a cryostat (Leica CM 3050S) at −21°C and stained for myofibrillar ATPase at pH 9.4 after pre‐incubation at pH 4.3. Type I fibres stained dark, type II fibres light and hybrid type I/II fibres intermediate (*Figure* *1* *A,B*).

### Morphometry

The stained cross sections were photographed under a light microscope (Carl Zeiss Vision GmbH, Aalen, Germany) at ×10 objective with a digital camera (Zeiss AxioCam MRc, Göttingen, Germany). The contours of each fibre were drawn using Btablet (https://hoofd.info/louis/s/s_Apps.html, Ooij, the Netherlands), and the coordinates of the outlines were stored for further analysis with AnaTis (https://hoofd.info/louis/s/s_Apps.html).

The mean number of fibres measured per biopsy was 139 (range 38–336) type I and 140 (range 37–367) type II fibres in 2002 and 164 (range 43–362) type I and 154 (range 52–412) type II fibres in 2012. The fibre type proportion (expressed as fibre number percentage) and the fibre cross‐sectional area (FCSA) of each fibre were calculated. The variation in FCSA was expressed as the standard deviation of the FCSA (SD FCSA). The shape factor of the fibre was calculated as follows:

$${\text{perimeter}}^{2}/\left(\text{4π}\times \text{FCSA}\right)$$

where an increased shape factor indicates increased angularity,^15^ suggesting denervation.

### Analysis of fibre type grouping

The proportion of type grouping in a cross section was assessed using the method of Jennekens et al.^17^ An ‘enclosed fibre’ was defined as any muscle fibre of a given type surrounded exclusively by fibres of the same type, and a fibre type group was defined as a group of fibres with at least one enclosed fibre.^15^, ^17^ In each cross section, the number of enclosed fibres for each type was counted manually. The prevalence of enclosed fibres (%), reflecting fibre type grouping, was calculated as

$$100\%\times n_{\text{enclosed}}/n_{\text{total}}$$

where ‘n_enclosed_’ is the number of enclosed fibres of a given type and ‘n_total_’ is the total number of muscle fibres of the same type in a region of interest.^17^

To determine the extent to which the muscle fibres were enclosed by chance, a mathematical model was used that has been applied previously to 36 different human muscles with a large difference in fibre type composition.^18^ The model assumes a random spatial arrangement of the fibres of the two main histochemical types (type I and type II fibres). Additionally, it is assumed that the proportion of type I fibres is constant throughout a cross section. The number of neighbours for each fibre in a cross section was counted. The prevalence of the expected fibre type grouping (%) was calculated as follows:

$$\%\;\text{Fibres}\;\text{of}\;a\;\text{given}\;\text{type}\;\text{enclosed}\;\mathrm{by}\;\text{chance}=100\times P^{n+1}$$

where ‘P’ is the proportion of a given fibre type in the cross section and ‘n’ is the number of fibres surrounding a fibre of a particular type in the cross section. Occasionally, enclosing fibres were on the edge of the region of interest and included as enclosing fibres.^15^

As there may be more than one fibre in a group surrounded by fibres of the same type, the percentage of enclosed fibres may overestimate the number of groups. Therefore, we also calculated the number of groups per 1000 fibres as

Numberofgroupsobserved*1000/numberoffibres

where the number of fibres and that of groups include fibres at the edge of the region of interest.

### Statistics

All data were analysed with IBM SPSS Version 27. A Kolmogorov–Smirnov test was used to test whether the data were normally distributed. If this was not the case, the data were log‐transformed before statistical analysis. Changes in height, body mass, BMI and fibre type composition over the 10 years were determined with a paired *t*‐test. Changes in FCSA, SD FCSA and shape factor were compared with a repeated‐measures analysis of variance (ANOVA) with as within factors fibre type (type I vs. type II) and time (2002 vs. 2012) and co‐variate ‘age in 2002’. Changes in group number between fibre types over the 10‐year period and whether it deviated from that expected were tested with a repeated‐measures ANOVA with the same factors as used for FCSA, but with the addition of expected versus observed grouping as within factor. Correlations were given as Pearson's correlation coefficients. Effects, interactions, changes and correlations were considered significant at *P* < 0.05.

## Results

### Participant characteristics

**Figure 2:**
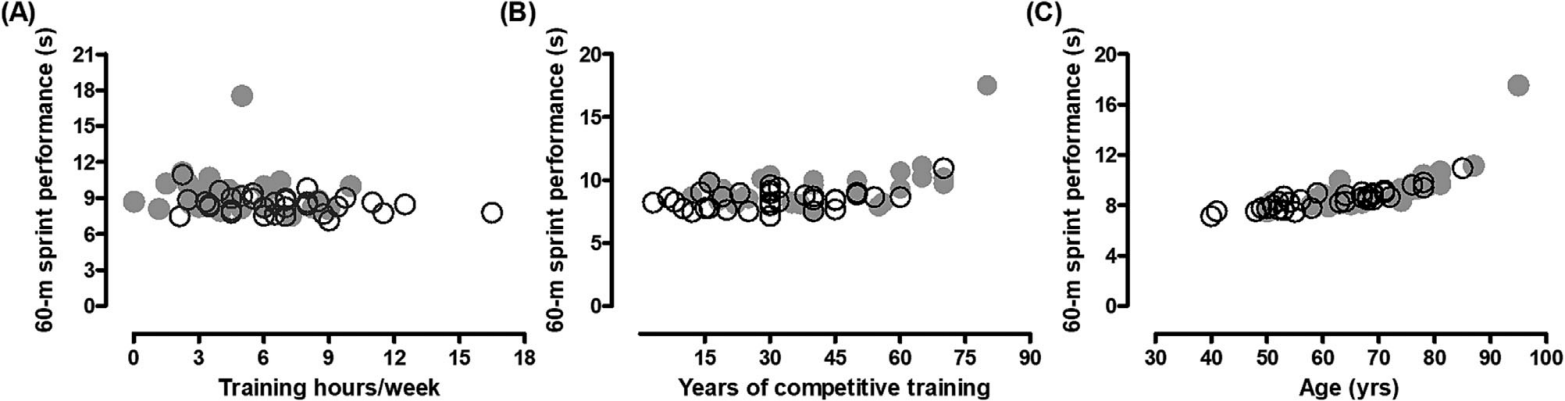
Correlation between 60‐m performance with (A) training hours per week, (B) years of competitive training and (C) age in 2002 (○) and 2012 (●). The outlier is the 85‐year‐old (in 2012, 95‐year‐old) person, but in Panel (C), his data point corresponds with the expected exponential age‐related decline in performance.

Although two participants had stopped competing in 2012, they were still training and therefore included for further analysis. In 2002, the youngest participant was 40 and the oldest participant was 85 years old. The athletes lost ~1 cm of height in the 10‐year period (*P* < 0.001), but there were no significant changes in body mass or BMI (*Table* *1*). The number of weekly training hours and 60‐m sprint performance were lower in 2012 than in 2002 (*P* < 0.001; *Table* *1*). There were no significant correlations between weekly training hours (*Figure* *2* *A*), or the number of years trained (*Figure* *2* *B*), with sprint performance. However, there was an exponential decrease in 60‐m sprint performance with age, where the follow‐up data overlapped the baseline data (*Figure* *2* *C*). The reduction in 60‐m sprint performance amounted to 1.10 ± 1.20 s (*n* = 27). However, the 85‐year‐old (in 2002) athlete showed a 6.6‐s increase in time needed to complete a 60‐m sprint. Excluding this athlete from the analysis still showed a slowing of the 60‐m sprint performance (*P* < 0.001). The age‐graded performance (AGP) of the athletes was 92% in both 2002 and 2012 (*Table* *1*), indicating a high‐performance status in this group.

**Table 1:** Participant (*n* = 34) characteristics in 2002 and 2012

	Age (years)	Height (m)	Body mass (kg)	BMI (kg·m^−2^)	Years trained	Training hours per week	60‐m sprint (s)	AGP (%)
2002	61.5 ± 10.9	1.75 ± 0.05	73.3 ± 7.8	23.9 ± 1.5	30.9 ± 16.2	6.84 ± 3.14	8.50 ± 0.79 (33)	92.4 ± 3.6 (33)
2012	71.5 ± 10.9*	1.74 ± 0.06*	73.0 ± 8.7	24.1 ± 1.8	42.0 ± 19.1*	4.43 ± 2.39*	9.48 ± 1.84 (28)*	92.5 ± 5.4 (28)

*Note*: Data are mean ± SD (*n*). Abbreviations: AGP, age‐graded performance; BMI, body mass index.

* Different from 2002 at *P* < 0.001.

### Fibre type composition, fibre cross‐sectional area and fibre size variation

Two biopsy cross sections of the myosin ATPase staining for the same athlete in 2002 and 2012 are shown in *Figure* *1* *A,B*. Hybrid fibres (intermediate staining intensity) were excluded from analysis as their proportion was <1% of the fibre population in all biopsies. No significant changes were noted in fibre type composition over the follow‐up period (*Figure* *1* *C*).

**Figure 3:**
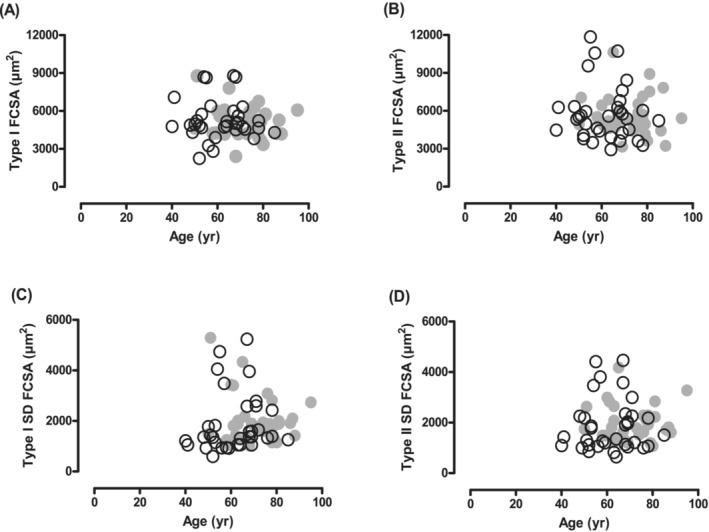
Relationship between age and fibre cross‐sectional area (FCSA) of (A) type I and (B) type II fibres, and standard deviation of the fibre cross‐sectional area (SD FCSA) fibres of (C) type I and (D) type II fibres in the vastus lateralis muscle of masters athletes in 2002 (○) and 2012 (●) (*n* = 34).

*Figure* *3* shows the FCSA and the variation in FCSA. There was no significant reduction in the FCSA (*Figure* *3* *A,B*), nor a significant increase in the fibre size variation, reflected by SD FCSA (*Figure* *3* *C,D*), in type I or type II fibres over 10 years.

### Fibre type grouping

As there were no significant changes in fibre type composition over the 10‐year period, and because we previously showed that there was no significant difference in fibre type grouping between type I and type II fibres,^15^ we have shown here data of all fibres pooled.

**Figure 4:**
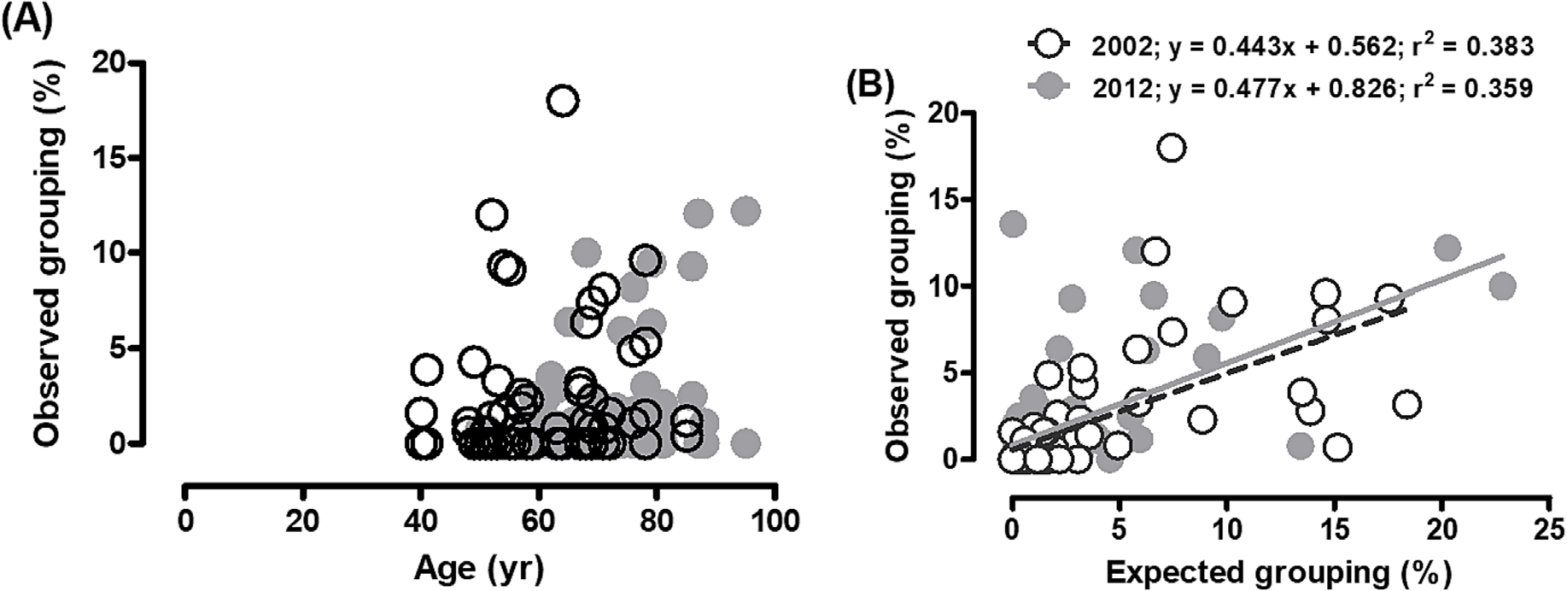
Relationship between observed fibre type grouping (A) with age and (B) expected fibre type grouping in the vastus lateralis muscle of masters athletes in 2002 (○) and 2012 (●) (*n* = 34). The regression lines for observed versus expected grouping in 2002 (solid line) and 2012 (dashed line) are similar.

**Figure 5:**
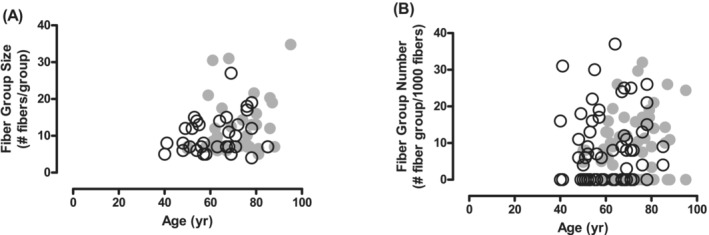
(A) Fibre group size and (B) number of fibre groups per 1000 fibres in the vastus lateralis muscle of masters athletes in 2002 (○) and 2012 (●) (*n* = 34).

No significant difference in the proportion of enclosed fibres was noted during the 10‐year follow‐up (*Figure* *4* *A*). The correlation between the observed and expected proportions of enclosed fibres was also similar in 2002 and 2012 (*Figure* *4* *B*). The fibre group size (*Figure* *5* *A*) and the number of fibre groups per 1000 fibres (*Figure* *5* *B*) did not change significantly over the 10‐year follow‐up period.

**Figure 6:**
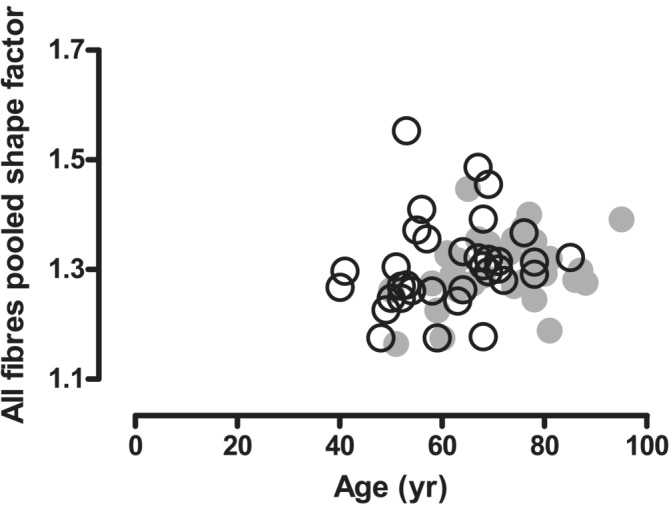
Relationship between age and shape factor of all pooled fibres in masters athletes in 2002 (○) and 2012 (●) (*n* = 34).

The deviation from the normal shape of a fibre is reflected by the shape factor. There was no significant change in shape factor over the 10‐year period (*Figure* *6*).

## Discussion

The major finding of the present longitudinal study on 40‐ to 85‐year‐old male sprinters is that over 10 years of continued sprint run training (50–95 years), there was neither significant fibre atrophy nor increased fibre type grouping, even though 60‐m sprint performance was reduced. These data support our earlier cross‐sectional observation showing no evidence of an ageing‐related increase in fibre type grouping in both athletes and non‐athletes.^15^ Taken together, these data suggest limited, or no, ageing‐related motor unit remodelling in either athletes or non‐athletes.

### Athletic performance

In 2002, the AGP for the 60‐m sprint of our athletes was 92%, showing their exceptional performance level. As reported in a previous bone study for the same athletes,^19^ the 60‐m sprint performance was reduced over the 10‐year follow‐up. It is interesting that the performance in the sprint was independent of the self‐reported number of years trained and the training hours per week, something also observed in swimmers where an increase in training volume did not result in improved performance.^20^ In the current study, the one person who at 10‐year follow‐up was an apparent outlier and had a lower performance than the other sprint runners was the oldest participant. This observation supports the suggestion that performance declines exponentially during ageing and accelerates in the oldest old athletes as we have reported previously with longitudinal records.^21^ The observation that the 10‐year follow‐up data for performance and all other parameters overlaid the baseline data indicates, as we showed before for performance in other track and field disciplines,^21^ that cross‐sectional data give a good indication of ageing‐related changes in performance.

### Fibre type composition and fibre size

Our longitudinal data support previous cross‐sectional observations in masters sprint runners^15^ and cyclists^22^ of no significant change in fibre type composition with age. Cross‐sectional studies have shown that the ageing‐related loss of muscle mass is at least partly attributable to type II fibre atrophy in healthy ageing individuals,^23^, ^24^, ^25^ where the absence of a significant fibre atrophy in ageing cyclists^22^ suggests that regular endurance training prevents this ageing‐related type II fibre atrophy. However, it has been reported that the fibre size of older endurance runners was less than that of young controls, while resistance‐trained older athletes exhibit a similar fibre size as the young controls.^26^ These observations may indicate that the type of overloading (muscle forces in cycling and weightlifting vs. gravity‐derived impacts during running) has a significant impact on fibre size.

The absence of significant atrophy in our masters sprint runners over a 10‐year period at first glance suggests that sprint training (i.e., running complemented with plyometric and strength exercises) does prevent muscle fibre atrophy. However, we previously found in a cross‐sectional study that the absolute rate of type II fibre atrophy was actually faster, rather than attenuated, in masters sprinters than non‐athletes, and hence, the difference in FCSA of type II fibres between masters athletes and non‐athletes decreased with increasing age, though the annual percentage of decline was similar.^15^ One could argue that the ageing‐related atrophy in masters sprinters in the cross‐sectional study was just a reflection of interindividual differences in genetic constitution. However, we consider this highly unlikely given that in 9‐ to 12‐year longitudinal studies in ageing non‐athletes, there was no significant muscle fibre atrophy.^27^, ^28^ Rather, the absence of significant muscle fibre atrophy in longitudinal studies may be attributable to a type II error, as fibre atrophy amounts to around 10% over 10 years, assuming a rate of 1% a year,^29^ which is smaller than the 16% standard deviation of type II fibre size.^27^ Therefore, overall, the pattern emerges of an ageing‐related type II muscle fibre atrophy, even in masters sprint runners.

### Fibre type grouping

Electrophysiological studies suggest a progressive loss of motor units during ageing starting at around the age of 50 years^4^, ^5^ that is thought to be attributable to motor neuron loss.^1^ The ageing‐related loss of motor units appears accompanied by an increase in motor unit size, based on an increase in individual motor unit potentials.^5^ The increased motor unit size has been suggested to reflect a reinnervation of fibres that became denervated consequent to the demise of their innervating motor neuron.^1^ This denervation–reinnervation process results in fewer but larger motor units. It has been hypothesized that the same process results in both an increased clustering of fibres of the same type (fibre type grouping) and an increased variation in fibre size. This has been observed more frequently in histological sections from muscles of people in their eighth decade compared with younger individuals.^30^ Histological sections are thus considered useful to assess (i) ageing‐related motor unit remodelling and (ii) whether this remodelling is attenuated by regular exercise training as exhibited by masters athletes.

In the current histological investigation, we found no significant increase in fibre type grouping in masters sprint runners over a 10‐year period of sustained training. This finding suggests that there was no significant motor unit remodelling during this 10‐year period, similar to what we previously observed in a cross‐sectional study in masters sprinters.^15^ The finding supports the notion that long‐term training attenuates motor unit remodelling.^7^

Previous cross‐sectional studies suggested that larger motor unit action potentials^8^ and a larger percentage of fibre type groups in older recreational sportsmen than untrained controls^9^ indicate enhanced reinnervation in athletes. Such an interpretation may, however, be premature as in our previous cross‐sectional study and the current 10‐year follow up study, we found no ageing‐related increase in fibre type grouping in either athletes or non‐athletes.^15^ Perhaps even more striking is a recent observation of less, rather than more, fibre type grouping in muscles from 70‐ than 21‐year‐old men, even though there was significant type II fibre atrophy.^25^

Previous histological studies reporting an ageing‐related increase in fibre type grouping in non‐athletes^30^, ^31^, ^32^ and athletes^9^ either described grouping qualitatively rather than quantitatively^30^, ^32^ or used a definition of grouping (fibres do not need to be surrounded exclusively by fibres of the same type)^9^, ^31^ more lenient than the conventional definition^17^ used in the present study. Importantly, in the study showing more fibre type grouping in older male athletes of diverse sports compared with non‐athletes, the type I grouping correlated significantly with the proportion of type I fibres.^9^ Given that (i) the observed prevalence of fibre type grouping did not differ significantly from that predicted from the fibre type composition in athletes, non‐athletes^15^ and rats,^33^ and (ii) the positive relationship between type I grouping in muscles from young and old men with the proportion of type I fibres,^25^ we suggest that the larger type I grouping in athletes than non‐athletes seen by Mosole et al.^9^ was a result of a larger proportion of type I fibres, rather than an improved reinnervation compared with the non‐athlete population. If the higher proportion of type I fibres in their athletes were the result of motor unit remodelling consequent to denervation–reinnervation, then such a type II to type I shift would be indicative of more denervation–reinnervation in the athletes than non‐athletes, as the non‐athletes showed no ageing‐related difference in fibre type composition from the young controls.^9^ We therefore propose that the higher proportion of type I fibres in the older athletes than the sedentary population^9^ is either related to a genetic predisposition to a higher proportion of type I fibres in their athletes and/or a response to the long‐term exercise programme where indeed fibre type shifts can be induced by, for example, repeated electrical stimulation,^34^ not involving any denervation–reinnervation.

Overall, combining the observations in our previous^15^ and other histological studies in athletes^9^ and non‐athletes^25^ with findings in our current investigation does not support the electrophysiological evidence for ageing‐related motor unit remodelling, let alone attenuated motor unit remodelling with long‐term physical training.

Denervation–reinnervation may also be reflected by the presence of angulated fibres. Therefore, the higher incidence of angulated fibres of muscle from non‐trained than long‐term trained older people^9^ appears to support the conclusion from electrophysiological experiments that long‐term endurance and power training facilitates motor unit expansion.^8^ The absence of a change in shape factor and variation in fibre sizes in the present 10‐year follow‐up study appears to support these observations that regular exercise attenuates denervation and/or stimulates reinnervation. However, in our previous cross‐sectional study,^15^ there was no ageing‐related increase in the variation of fibre sizes or shape factor in non‐athletes, suggesting that there is no significant difference in motor unit remodelling—if it occurs at all—between athletes and non‐athletes.

It should be noted that also the electrophysiological observations are equivocal. For instance, it has been observed that both motor unit size and muscle size were larger in the athletes than non‐athletes, or the tennis than non‐tennis arm.^8^ Therefore, the larger motor unit size may be simply the result of larger, rather than more, fibres per motor unit. In addition, while motor unit size determined with intramuscular electromyography (EMG) was higher in the vastus lateralis of endurance and power athletes than non‐athletes, and in the favoured arm of tennis players, no such motor unit expansion was seen when derived from the surface EMG.^8^ Thus, if only surface EMG data were available, the conclusion of that study would have been that long‐term training does not facilitate motor unit size expansion. The question therefore emerges whether training does, as suggested in the literature, facilitate reinnervation or not.

In summary, both our current longitudinal and previous cross‐sectional studies in masters athletes and non‐athletes^15^ show no histological evidence of ageing‐related motor unit remodelling, neither in terms of grouping nor in terms of changes in fibre shape and size distribution, or fibre type composition. This challenges the concept of motor unit remodelling during ageing. As discussed previously,^4^, ^15^ histological sections cover a small muscle area while during electrophysiological measurements, a larger muscle volume is sampled, and hence, histological analysis may not provide an adequate indication of motor unit remodelling during ageing. While this is a potential drawback of histological evaluation of motor unit remodelling, the similar fibre type composition and overlapping fibre sizes at baseline and 10 years later, combined with the virtually identical correlation between observed and expected fibre type grouping, indicate that what we observed is representative of the morphology of the vastus lateralis muscle, at least for the region where the biopsies were taken.

Conceivably, part of the explanation for the discrepancy between our histological evidence and the often‐seen electrophysiological evidence for an ageing‐related motor unit remodelling is that motor unit remodelling does not need to result in fibre type grouping. Indeed, in rat muscles, an ageing‐related increase in motor unit size with fibres spread over a larger territory was previously observed without evidence of fibre type grouping.^35^ This, and the fact that grouping appears to be related to the fibre type composition, suggests that one must be careful to interpret fibre type grouping as evidence for denervation–reinnervation.

Perhaps we also need to reconsider whether the electrophysiologically deduced motor unit remodelling during ageing^4^, ^5^ is indeed a reflection of denervation–reinnervation and/or the result of other processes. For example, changes in the relationship between motor unit action potential and the force generated, and prolongation of the action potential have been reported in rat^36^ and human^4^, ^37^, ^38^ muscles during ageing. As motor unit size is often given as the time integral of the negative peak of the action potential, a prolonged action potential per se can be wrongly interpreted as an increase in the motor unit size. An increased duration of the action potential during ageing has previously been suggested to come from a higher fibre density,^38^ slower conduction velocity (e.g., due to fibre atrophy) and/or a larger dispersion of fibres of a motor unit^36^ that may be associated with motor unit remodelling. However, they have also been shown to be associated with an increased chloride ion channel conductance^39^ that is independent of motor unit remodelling.

We suggest that to answer the question whether electrophysiological and histological analyses can indeed indicate motor unit remodelling during ageing, longitudinal studies that assess both histological fibre type grouping, and electrophysiological motor unit number and motor unit size are required. Our study was a first step in that direction and revealed that in sprint‐trained athletes, there is no evidence for motor unit remodelling, and any fibre type grouping present was not different from that predicted from the fibre type composition.

## Conclusions

Our 10‐year follow‐up study in male masters sprinters (50‐ to 95‐year‐old at the end of the study) showed no evidence for motor unit remodelling. It confirms our previous cross‐sectional observation of an absence of an ageing‐related increase in fibre type grouping, despite significant type II fibre atrophy.^15^ Taken together, the findings in these studies challenge the notion that ageing is associated with significant motor unit remodelling.

## Funding

Juho Vainio Foundation; Opetus‐ ja kulttuuriministeriö; YrjöJahnsson Foundation; Päivikki and Sakari Sohlberg Foundation; European Union.

## Conflict of interest statement

The authors declare no conflicts of interest.
